# Conditioned plasma promotes full-thickness skin defect healing in a rat model

**DOI:** 10.1016/j.reth.2025.08.003

**Published:** 2025-08-18

**Authors:** Majid Zamani, Saeid Kaviani, Mehdi Yousefi, Saeid Abroun, Mohammad Hojjat-Farsangi, Behzad Pourabbas

**Affiliations:** a Department of Hematology, Faculty of Medical Sciences, Tarbiat Modares University, Tehran, Iran; b Immunology Research Center, Tabriz University of Medical Sciences, Tabriz, Iran; c Bioclinicum, Department of Oncology-Pathology, Karolinska Institute, Bioclinicum, Stockholm, Sweden; d Department of Polymer Engineering, Sahand University of Technology, Tabriz, Iran

**Keywords:** Conditioned plasma, Platelet rich plasma, Growth factor, Wound healing, Regenerative medicine

## Abstract

**Introduction:**

Blood derivatives may enhance wound healing, but each possesses distinct characteristics and has yielded varying outcomes in patient treatment. This research seeks to examine the efficacy of conditioned plasma (CP) using polylactic acid (PLA) coated beads and to compare it with CP using bare beads and platelet-rich plasma (PRP) in the context of acute wound healing.

**Methods:**

Blood was collected from 7 volunteer donors in three tubes containing ACD anticoagulant, PLA coated, or bare beads and incubated for 6 h at 37 °C. The concentration of VEGF, PDGF, TGF-β, IL-1β, IL-13, and IL-1Ra were measured by ELISA. Full-thickness wounds were made on the back of rats. PRP, CP with PLA-coated bead or bare beads, and phosphate buffer saline as control were administered to the wound area. Wound closure rate at days 3, 7, 10, and 14; epithelialization, fibroblast cells, inflammatory cells infiltration, new collagen formation, new vessel, and immunohistochemistry (CD31, α-SMA) were measured 14 days after the incision.

**Results:**

The concentration of VEGF, PDGF, TGF-β, IL1-β, and IL-1Ra was significantly higher in CPs than in PRP (*p* < 0.05). CP with PLA-coated beads promoted wound closure and improved skin wound healing (*p* < 0.05), which was associated with enhanced epithelialization, fibroblast cell proliferation, new collagen formation, and reduced inflammatory cells infiltration. Immunohistochemistry showed an increase in CD31 and α-SMA levels in the treatment groups compared to the control group, but this increase was insignificant (*p* > 0.05).

**Conclusion:**

CP promotes wound healing by increasing epithelialization, fibroblast proliferation, collagen synthesis and deposition, and reducing inflammatory cells infiltration.

## Graphical abstract

Comparison of the effect of conditioned plasma and platelet rich plasma on wound healing in an animal model.

**Figure:**
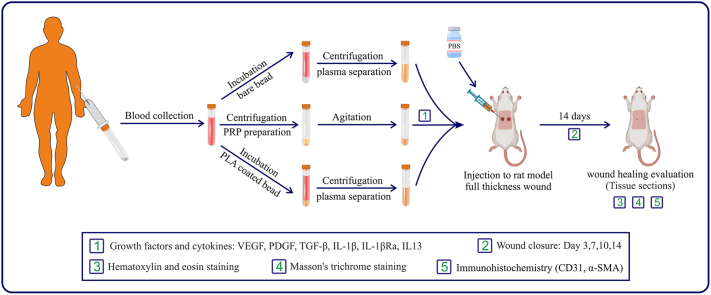

## 1 Introduction

Wound healing is a complex, multi-step process that includes hemostasis, inflammation, growth, re-epithelialization, and remodeling, and these steps can overlap [1,2]. Disrupting these processes might cause the wound to become chronic, take longer to heal, and leave a scar. In addition to burdening national health systems, prolonged and chronic wound healing can cause patients to have aesthetic, psychological, and financial issues [1,3]. Different cells play a role in the progress of wound healing phases. Platelets are the first cells that reach the injured site. By secreting various compounds, platelets help prevent bleeding, call and activate other cells, and secrete growth factors and cytokines to promote tissue repair. Different types of immune cells, such as neutrophils, monocytes, and macrophages, are present at the injury site and play a role in preventing infection and progressing wound healing [4,5]. These cells and their secreted factors are present in blood derivatives. The use of blood derivatives has attracted much attention in the last decade. Various types of blood derivatives, such as platelet rich plasma (PRP), platelet lysate (PL), and autologous conditioned serum (ACS), are used in cell proliferation, regenerative medicine, and wound healing [[6], [7], [8], [9]].

PRP is a high-concentration source of autologous platelets, containing compounds and micronutrients, growth factors, and cytokines such as platelet-derived growth factor (PDGF), transforming growth factor-beta (TGF-β), and vascular endothelial growth factor (VEGF) that can play a role in cell proliferation, angiogenesis, and wound healing [10,11]. Platelet lysate includes chemicals within the platelets and is created when the platelet membrane is destroyed by thrombin, ultrasonication, and freeze/thaw [12]. ACS is another blood derivative produced by activating cells, mainly monocytes, using borosilicate glass beads. ACS contains different type of cytokines, such as interleukin (IL)-1β, IL-6, IL-1 receptor antagonist (IL-1Ra), and IL-10, used mainly in osteoarthrosis [13]. A study demonstrated that ACS accelerated wound healing in diabetic mice by stimulating fibroblast proliferation and inhibiting the stimulator of interferon genes (STING) pathway [14]. In a separate study, ACS dressings were applied to hard-to-heal wounds, revealing that this blood derivative reduced wound surface area and enhanced the healing process [8]. Along with glass beads, substances like collagen, carbon, and polylactic acid are employed to stimulate cells [[15], [16], [17], [18]]. In a study, glass beads coated with nanocarbon materials to activate cells and their effect on knee osteoarthritis was performed [19]. The study's results showed an increase in the concentration of cytokines and growth factors in the ACS and better effectiveness in treating patients.

Polylactic acid (PLA) has been used in nanomedicine as drug delivery systems, cancer chemotherapy, gene delivery constructs, growth factor carriers, antibacterial agents, magnetic resonance imaging, scaffolds, skin rejuvenation, wound healing, and bone repair [18,20,21]. Considering the biological properties of nanoparticles with biodegradable capability, the US Food and Drug Administration (FDA) has approved the use of these nanoparticles, including PLA nanoparticles (PLA-NP), in humans [20,22]. Numerous investigations of PLA-NP's harmful effects on cell lines have been carried out, showing that the nanoparticles are not dangerous to cells. However, depending on the nanoparticles' size, shape, morphology, and surface charge, their use may have varying adverse impacts [23,24]. These side effects include reactions with proteins, carbohydrates, and other micro molecules, activation of immune cells, especially macrophages and monocytes, changes in cell metabolism, and alterations in the activity of platelets and coagulation proteins [17,18,23,[25], [26], [27]]. However, the exact mechanism of immune activation of these nanoparticles is still poorly understood [18,28]. Studies have found nanoparticles with minimal side effects for therapeutic purposes, but these side effects of PLA-NP can stimulate cells to produce cytokines and growth factors.

In this study, we intend to investigate the effect of borosilicate beads coated with PLA-NP on the concentration of growth factors and cytokines in conditioned plasma (CP) and its effectiveness on microscopic and macroscopic wound healing in rat animal models.

## 2 Materials and methods

### 2.1 Blood collection and blood derivatives preparation

Human blood samples were withdrawn from healthy donors following regional guidelines and approval from the Ethics Committee of Tarbiat Modares University (IR.MODARES.AEC.1403.011(. After a full explanation of the study conditions and blood collection, written informed consent was obtained from the donors for using their whole blood for research purposes. The volunteers had no underlying disease, platelets less than 150 × 10^3^/μL, platelet disorders, hemoglobin less than 12 g/dL, immunosuppressive drugs, blood malignancy, white blood cells (WBCs) less than 6 × 10^3^/μL, or smoking.

**Fig. 1:**
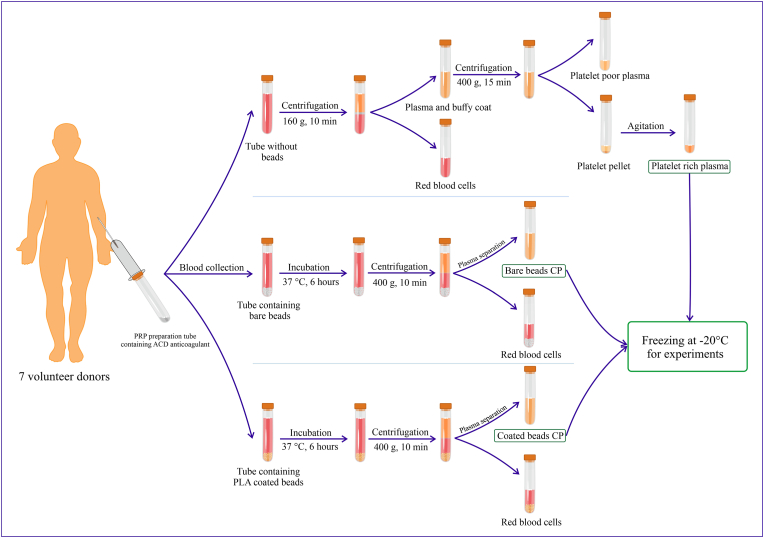
**Procedure for the preparation of blood derivatives**. Blood was collected from seven healthy volunteers into tubes containing acid citrate dextrose (ACD) anticoagulant. For platelet-rich plasma (PRP) preparation, whole blood was centrifuged in a tube without beads to separate the plasma and buffy coat. The plasma was then centrifuged again to pellet the platelets, and the platelet-poor plasma (PPP) was discarded. The platelet pellet was resuspended in residual plasma to obtain PRP. For conditioned plasmas (CPs) preparation, whole blood was incubated in tubes containing either polylactic acid (PLA) coated beads or bare beads for 6 h at 37 °C and subsequently centrifuged to isolate the plasma. All derivatives were stored at −20 °C until further use.

For the preparation of PRP, 25.5 ml of whole blood was drawn from 7 volunteer donors across three tubes, each tube containing 8.5 ml of whole blood and 1.5 ml of acid citrate dextrose (ACD) anticoagulant (BD Vacutainer® PRP Preparation Tube). The procedures for preparing PRP and CPs are illustrated in Fig. 1. Whole blood was collected to prepare PRP and CPs using bare beads and PLA-coated beads [29]. No compound was added to the PRP preparation tube; 33 bare beads with a diameter of 3 mm were placed in one tube, and 33 beads with a diameter of 3 mm coated with polylactic acid nanoparticles were placed in the other tube [8,19].

A two-stage centrifugation method was used to prepare PRP [30]. First, the blood sample was centrifuged for 10 min at 160 g. The supernatant was separated and transferred to another tube. In the next step, it was centrifuged for 15 min at 400 g. Two-thirds of the supernatant, platelet-poor plasma (PPP), was discarded, and the remaining one-third of the plasma, along with the platelets, was separated as PRP. The samples were subjected to continuous gentle agitation for 1 h to homogenize.

Two tubes containing PLA-coated beads or bare beads were incubated at 37 °C for 6 h [8,19]. After incubation, the tubes were centrifuged at 400 g for 10 min, and the plasma was separated. 400 μL were separated from each tube for enzyme-linked immunosorbent assay (ELISA) tests, and the remaining samples of each blood derivative were pooled. All blood derivatives were stored at −20 °C for subsequent experiments.

### 2.2 Growth factors and cytokine measurement

The concentration of growth factors and cytokines in the blood derivatives produced in all three groups was measured by the ELISA technique. For this purpose, the samples were first thawed at 4 °C. After thawing, the samples were centrifuged for 15 min at 1000 g, and the growth factors and cytokines TGF-β, VEGF, PDGF, IL-1β, IL-13, and IL-1Ra (MyBiosource, USA), were measured according to the manufacturer's instructions.

### 2.3 Experimental animals

All procedures for animal studies were approved by the Ethics Committee of Tarbiat Modares University (IR.MODARES.AEC.1403.011(, and were carried out following the guidelines for handling laboratory animals. All experiments involving animals were conducted in accordance with the ARRIVE guidelines [31] to ensure rigorous and transparent reporting of animal research. Twenty 6-8 weeks-old male rats weighing 180–250 gr were purchased from the Animal Care Center of Tarbiat Modares University. Throughout the study, animals were kept in individual cages at the Animal Care Center of Tarbiat Modares University at 22 ± 1 °C, under a 12/12 h light/dark cycle, with free access to food and water.

### 2.4 Wound model and treatment protocol

Rats were randomly divided into four groups (n = 5 each): phosphate-buffered saline (PBS) (Dena Zist Asia, Iran) control, PRP, bare beads CP, and PLA-coated beads CP. Under anesthesia (ketamine (80 mg/kg body weight, Duga Ilac, Turkey) and xylazine (10 mg/kg body weight, AdvaCare Pharma, USA)), two full-thickness 8 mm excisional wounds were created on the dorsum using a sterile biopsy punch (Paramount Surgimed). Each rat received a 0.4 ml subdermal injection of its respective treatment around the wound. Post-surgical care included buprenorphine (0.1 mg/kg) and sterile dressings (Comfeel, Denmark) changed on days 3, 7, and 10.

### 2.5 Wound closure analysis

Wound closure was assessed by macroscopic analysis of the wound lesion area on rats' dorsal, days 3, 7, 10, and 14 after surgery. After photographing the wound, the wound area was calculated using ImageJ software v1.8.0. The rate of wound area was calculated using the following formula [32]:

$$W_{\%}=\left(W_{t}\;/W_{0}\right)\times 100$$

W%: Percent of wound area.

W_0_: **I**nitial wound area (day 0)

W_t_: The residual wound area (day X)

### 2.6 Histological observation

On day 14, rats were euthanized, and wound tissues were harvested and fixed in 4 % paraformaldehyde. After paraffin embedding and sectioning (4 μm thick), slides were stained with hematoxylin (Bahar Afshan, Iran) and eosin (Bahar Afshan, Iran) (H&E) and Masson's trichrome (Asia Pajohesh, Iran). Light microscopy (Olympus) was used for scoring of parameters like inflammatory infiltration, re-epithelialization, fibroblast activity, neovascularization, and collagen synthesis (Table 1) [33]. During wound healing, both new blood vessel formation and inflammatory cell infiltration gradually decrease over time [4,33,34]. Since lower levels of these markers indicate better healing progress, we inverted their scoring values for standardization, enabling calculation of a total score. This scoring system assigns higher values to wounds with less inflammatory cell infiltration and fewer new vessels.

**Table 1:** **Wound healing scoring system.** Hematoxylin and Eosin, and Masson's trichrome staining were used to score wound healing based on epithelialization, fibroblast proliferation, new collagen fiber formation, new vessels, and inflammatory cell infiltration.

Epithelization	Fibroblasts	Collagen	New vessels	Inflammatory cells
Thickness of cut edges = 0	Absent = 0	Absent-granulation tissue = 0	Marked = 0	Marked (over 75 %) = 0
Migration of cells (<50 %) = 1	Mild-surrounding tissue = 1	Minimal-granulationtissue = 1	Moderate = 1	Moderate (50–75 %) = 1
Migration of cells (≥50 %) = 2	Mild-granulation tissue = 2	Mild-granulation tissue = 2	Mild to moderate tissue = 2	Mild to moderate (25–50 %) = 2
Bridging the excision = 3	Moderate-granulation tissue = 3	Moderate-granulation tissue = 3	Mild = 3	Mild (Less than 25 %) = 3
Keratinization = 4	Marked-granulation tissue = 4	Marked-granulation tissue = 4	Absent = 4	Absent = 4

### 2.7 Immunohistochemistry staining

For CD31 and alpha-smooth muscle actin (α-SMA) immunohistochemical staining, at first, the paraffin sections were dewaxed and rehydrated by xylene and gradient alcohol. Then, treatment was performed with bovine serum albumin (Sigma-Aldrich, USA) and antigen retrieval. After inactivating endogenous peroxidases with H_2_O_2,_ the sections were incubated with primary antibody (Biorbyt, USA) at room temperature. Then, they were incubated with a secondary antibody, colored by diaminobenzidine solution (Sigma-Aldrich, USA). After washing, sections were counterstained with hematoxylin (Bahar Afshan, Iran). The samples were evaluated blindly by two pathologists using a light microscope. The evaluation was performed according to Table 2 with proportion and intensity scores using the modified Allred scoring system [35]. Additionally, the percentage of positively stained areas for CD-31 and α-SMA in immunohistochemical staining was quantitatively analyzed using ImageJ software (version 1.8.0).

**Table 2:** **Modified Allred scoring system.** The modified Allred Score system was used to analyze immunohistochemical staining of CD31 and alpha-smooth muscle actin (α-SMA) markers, and they were scored based on the Proportion and intensity of staining.

Proportion score (P)	Intensity score (I)	Allred score (P + I)
No cells are immunoreactive = 0	Negative = 0	Negative = 0-1
≤1 % of cells are immunoreactive = 1	Weak = 1	Weak = 2-3
1–10 % of cells are immunoreactive = 2	Intermediate = 2	Moderate = 4-6
11–33 % of cells are immunoreactive = 3	Strong = 3	Strong = 7-8
34–66 % of cells are immunoreactive = 4	–	–
67–100 % of cells are immunoreactive = 5	–	–

### 2.8 Statistical analysis

All quantitative data are presented as the mean ± SD. Statistical analysis was performed using GraphPad Prism v9.0. The one-way ANOVA test was used for parametric data, and the Kruskal-Wallis test was used for non-parametric data analysis. A *p-value* < 0.05 was considered statistically significant. In all tables and figures, we used ∗ to denote *p*-values. No significance = *p* > 0.05, ∗ = *p* < 0.05, ∗∗ = *p* < 0.01, ∗∗∗ = *p* < 0.001, and ∗∗∗∗ = *p* < 0.0001.

## 3 Results

### 3.1 Growth factors and cytokines

**Fig. 2:**
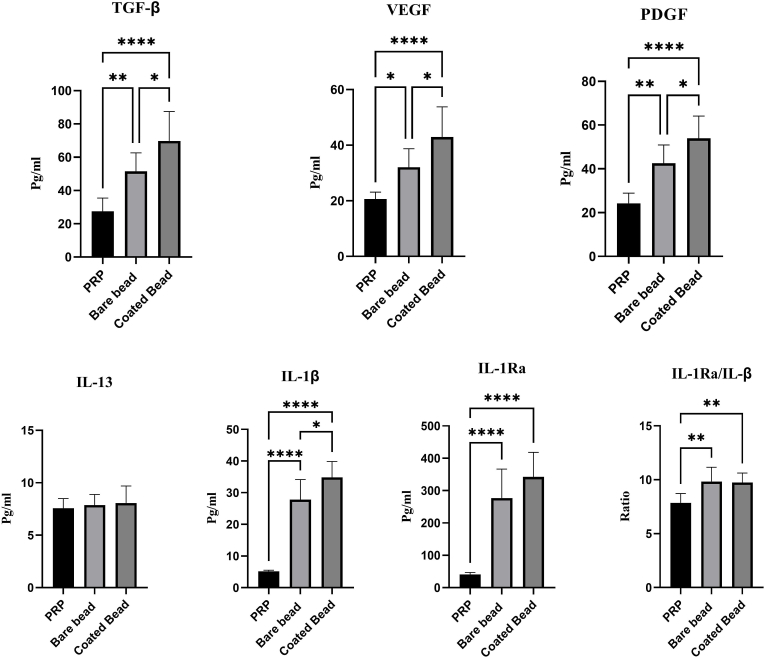
**Growth factors and cytokines concentration present in blood derivatives**. The graph represents the vascular endothelial growth factor (VEGF), Platelet-derived growth factor (PDGF), transforming growth factor beta (TGF-β), Interleukin 13 (IL-13), Interleukin 1 beta (IL-1β), Interleukin 1 receptor antagonist (IL-1Ra), and ratio IL-1Ra/IL-1β levels in platelet rich plasma (PRP), conditioned plasma with bare beads (bare beads CP), and conditioned plasma with PLA coated beads (coated bead CP). Data are presented as mean ± SD of seven donors. (∗*p* < 0.05, ∗∗*p* < 0.01, ∗∗∗*p* < 0.001, ∗∗∗∗*p* < 0.0001).

TGF-β, PDGF, VEGF, IL-1β, IL-1Ra, and IL-13 were measured using the ELISA method. The concentrations of growth factors and cytokines are listed in Table 3. The concentrations of growth factors (pg/ml) in the PRP, bare bead CP, and coated bead CP groups were as follows: VEGF levels measured 20.62 ± 2.52, 32.05 ± 6.67, and 42.91 ± 10.88; PDGF levels were 24.21 ± 4.71, 42.59 ± 8.28, and 53.95 ± 10.13; and TGF-β concentrations were 27.54 ± 7.93, 51.54 ± 11.10, and 69.79 ± 17.66, respectively. In addition, the concentrations of cytokines (pg/ml) in the PRP, bare bead CP, and coated bead CP groups were as follows: IL-13 levels measured 7.56 ± 0.92, 7.87 ± 1.00, and 8.067 ± 1.62; IL-1β levels were 5.17 ± 0.34, 27.80 ± 6.36, and 34.85 ± 4.99; and IL-1Ra concentrations were 40.83 ± 6.22, 276.7 ± 89.72, and 342.5 ± 75.55, respectively. The IL-1Ra/IL-1β concentration ratio in the PRP, bare bead CP, and coated bead CP groups was 7.86 ± 0.85, 9.83 ± 1.32, and 9.74 ± 0.88, respectively, indicating a higher level of the anti-inflammatory cytokine IL-1Ra to the inflammatory cytokine IL-1β in CPs. The results showed a higher concentration of TGF-β, PDGF, VEGF, IL-1β, and IL-1Ra in the coated bead CP and bare bead CP compared to PRP (*p* < 0.0001). However, the increase in IL-13 concentration was not significant in any of the groups (*p* > 0.05). In addition, the increase in cytokines TGF-β, PDGF, VEGF, and IL-1β was significant in the coated bead CP compared to the bare bead CP (*p* < 0.05) (Fig. 2).

**Table 3:** **Growth factor levels presented in PRP, bare bead CP, and coated bead CP. TGF-β, PDGF, VEGF, IL-1β, IL-13, and IL-1Ra** levels in PRP, bare bead CP, and coated bead are represented. Values are expressed as mean ± SD of seven donors. Statistically significant differences are shown in bold.

**Groups**→	Platelet rich plasma (a)	Bare bead CP a (b)	Coated bead CP (c)	*p value*		
↓**Growth factors and cytokine**				(a,b)	(a,c)	(b,c)
**VEGF**^b^ (Pg/ml)	20.62 ± 2.52	32.05 ± 6.67	42.91 ± 10.88	**0.0274**	**<0.0001**	**0.0368**
**PDGF**^c^ (Pg/ml)	24.21 ± 4.71	42.59 ± 8.28	53.95 ± 10.13	**0.0012**	**<0.0001**	**0.0413**
**TGF-β**^d^ (Pg/ml)	27.54 ± 7.93	51.54 ± 11.10	69.79 ± 17.66	**0.0071**	**<0.0001**	**0.0412**
**IL-13** (Pg/ml)	7.56 ± 0.92	7.87 ± 1.00	8.067 ± 1.62	0.8856	0.7282	0.9534
**IL**^e^**-1β** (Pg/ml)	5.17 ± 0.34	27.80 ± 6.36	34.85 ± 4.99	**<0.0001**	**<0.0001**	**0.0116**
**IL-1Ra**^f^ (Pg/ml)	40.83 ± 6.22	276.7 ± 89.72	342.5 ± 75.55	**<0.0001**	**<0.0001**	0.1933
**IL-1Ra/IL-1β**	7.86 ± 0.85	9.83 ± 1.32	9.74 ± 0.88	**0.0064**	**0.0091**	0.9854

a Conditioned plasma.

b Vascular endothelial growth factor.

c Platelet-derived growth factor.

d Transforming growth factor beta.

e Interleukin.

f Interleukin 1-β receptor antagonist.

### 3.2 Wound closure

**Fig. 3:**
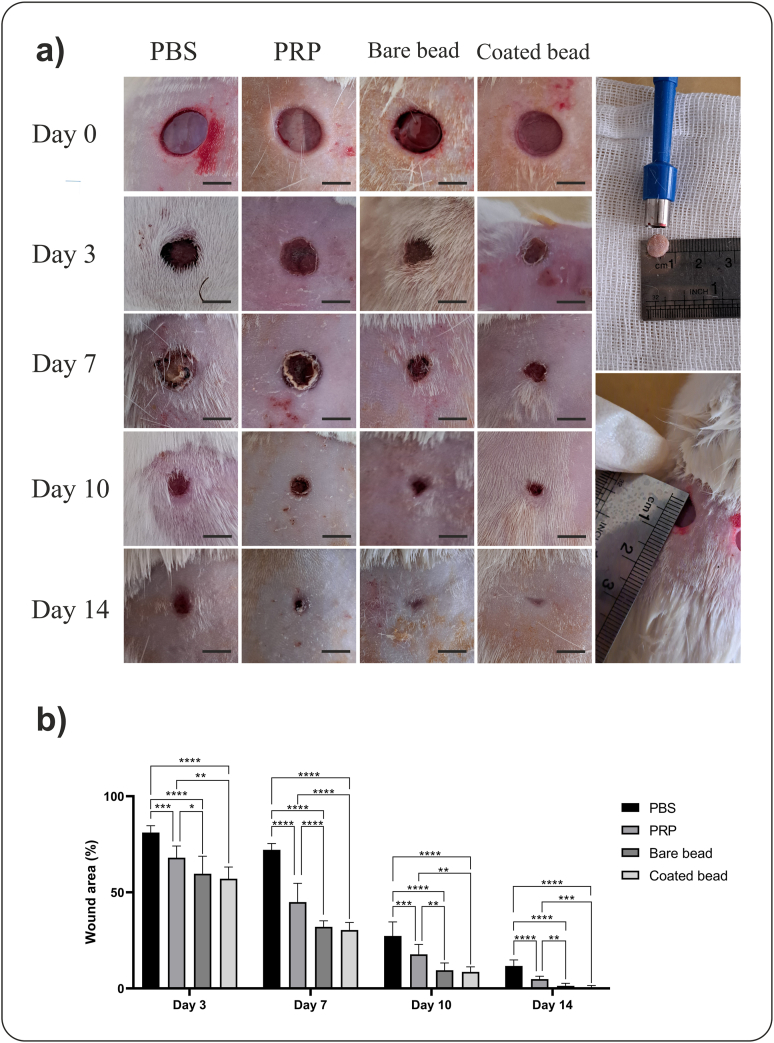
**Macroscopic analysis of wound healing**. Conditioned plasma (CP) accelerates wound closure in a rat full-thickness excisional wound model. The rats' hair was shaved on different days to visualize the wounds better. a) Full-thickness wound images on days 0, 3, 7, 10, and 14 after wounding in the phosphate-buffered saline (PBS), platelet-rich plasma (PRP), bare beads CP, coated beads CP treatment groups, indicating better wound closure in the CPs groups (scale bars = 5 mm). The two images on the right depict the excisional wounds in rats immediately after creation (Day 0). b) The wound area percentage. The CPs treated group exhibited significantly accelerated wound closure compared to the other groups. Data are presented as mean ± SD of full-thickens wounds of 5 rats per group and two excisional wounds per rat (n = 10 wounds per group). Statistical analysis: ∗*p* < 0.05, ∗∗*p* < 0.01, ∗∗∗*p* < 0.01, ∗∗∗∗*p* < 0.0001.

A full-thickness wound model was used to investigate the therapeutic effect of blood derivatives. Wound closure was evaluated on days 3, 7, 10, and 14 after treatment in all groups and compared with day 0 (Fig. 3a). The rats' hair was shaved on different days to visualize the wounds better. The average remaining wound area in the PBS, PRP, bare bead CP, and coated bead CP groups was 81.14 ± 3.57, 68.00 ± 6.14, 59.70 ± 9.09, and 57.08 ± 6.12 on day 3, 72.13 ± 3.30, 44.96 ± 9.77, 32.08 ± 3.10, and 30.39 ± 3.93 on day 7, 27.27 ± 7.39, 17.75 ± 5.19, 9.45 ± 3.84, and 8.60 ± 2.64 on day 10, 1.68 ± 3.17, 4.87 ± 1.51, 1.32 ± 1.33, 0.65 ± 0.57 on day 14, respectively. The rate of wound closure was higher in the groups treated with CPs, the increase in wound closure on all days was significant in the CPs groups compared to the control and PRP groups (*p* < 0.05), and this increase was also significant in the PRP-treated group compared to the control group (*p* < 0.001) (Fig. 3b).

### 3.3 Histopathological evaluation

**Fig. 4:**
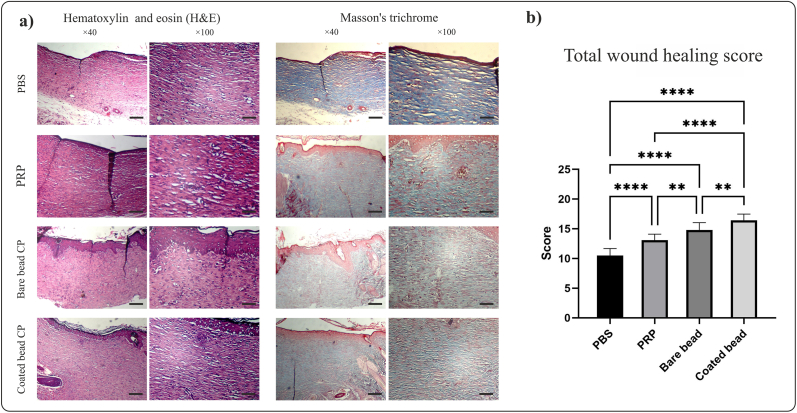
**Histological analysis of tissue sections at day 14 post wounding from full-thickness wounds in rat**. Conditioned plasma (CP) accelerates wound healing in a rat full-thickness excisional wound model. a) Image of hematoxylin and eosin (H&E) and Masson's trichrome staining in different treatment groups, phosphate buffered saline (PBS), platelet-rich plasma (PRP), bare beads CP, coated beads CP (magnification × 40, scale bars = 200 μm; magnification × 100, scale bars = 100 μm). b) Total score from wound healing parameters. Total scores of wound healing indicating better wound healing in the group treated with coated beads CP. Data are presented as mean ± SD of full thickens wounds of 5 rats per group and two excisional wounds per rat (n = 10 wounds per group). Statistical analysis: ∗*p* < 0.05, ∗∗*p* < 0.01, ∗∗∗*p* < 0.01, ∗∗∗∗*p* < 0.0001.

**Fig. 5:**
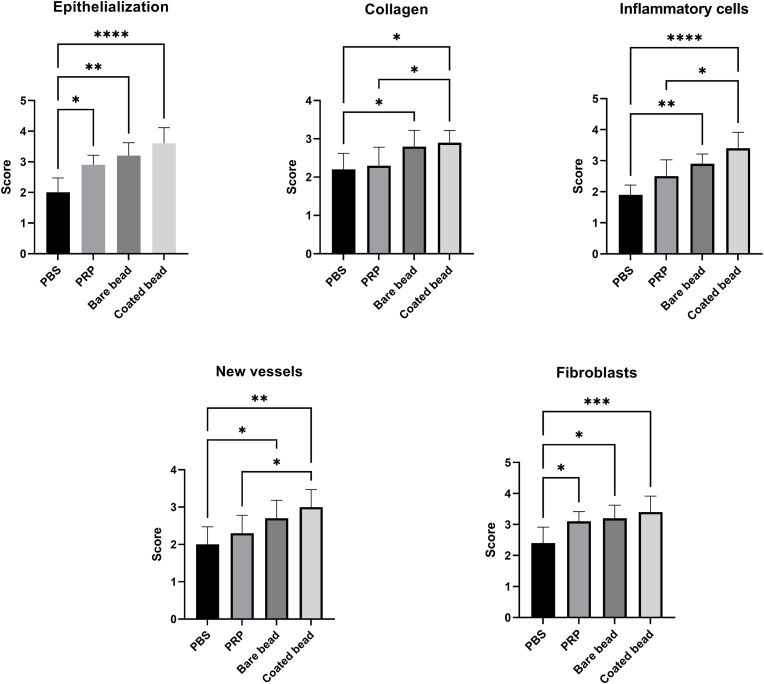
**Scoring of hematoxylin and eosin (H&E) and Masson's trichrome stained tissue sections**. Various parameters were evaluated: epithelialization, inflammatory cells infiltration, new vessels, fibroblasts, and new collagen formation Data are presented as mean ± SD of full thickens wounds of 5 rats per group and two excisional wounds per rat (n = 10 wounds per group). Statistical analysis: ∗*p* < 0.05, ∗∗*p* < 0.01, ∗∗∗*p* < 0.01, ∗∗∗∗*p* < 0.0001.

After preparing tissue sections and staining with H&E and Masson's trichrome (Fig. 4), different parts of the wound healing were scored (Table 4, Fig. 5).

**Table 4:** **Wound healing scoring in tissue sections in Hematoxylin and Eosin, Masson's trichrome, and immunohistochemistry stains.** Data are presented as mean ± SD of full thickens wounds of 5 rats per group and two excisional wounds per rat (n = 10 wounds per group).

Variables	PBS a	PRP^b^	Bare bead CP^c^	Coated bead CP
**Hematoxylin and Eosin (H&E)**				
Epithelialization	2.00 ± 0.47	2.90 ± 0.31	3.20 ± 0.42	3.60 ± 0.51
Inflammatory cells infilteration	1.90 ± 0.31	2.50 ± 0.52	2.90 ± 0.31	3.40 ± 0.51
New vessels	2.00 ± 0.47	2.30 ± 0.48	2.70 ± 0.48	3.00 ± 0.47
Fibroblasts	2.40 ± 0.51	3.10 ± 0.31	3.20 ± 0.42	3.40 ± 0.51
Collagen	2.20 ± 0.42	2.30 ± 0.48	2.80 ± 0.42	2.90 ± 0.31
All score	10.50 ± 1.17	13.10 ± 0.99	14.80 ± 1.22	16.30 ± 1.05
**Immunohistochemistry (CD31)**				
Proportion score	1.70 ± 0.67	2.40 ± 1.26	2.60 ± 0.84	2.60 ± 0.84
Intensity score	1.60 ± 0.69	1.60 ± 0.51	1.60 ± 0.84	1.90 ± 0.87
Allred score (P + I)	3.30 ± 1.33	4.00 ± 1.76	4.20 ± 1.54	4.80 ± 1.39
Percentage of stained areas	6.49 ± 4.65	11.70 ± 6.89	13.80 ± 7.02	15.60 ± 7.97
**Immunohistochemistry (α-SMA**^d^**)**				
Proportion score	3.20 ± 0.42	3.20 ± 0.42	3.40 ± 0.51	3.40 ± 0.51
Intensity score	2.20 ± 0.42	2.40 ± 0.51	2.40 ± 0.51	2.60 ± 0.51
Allred score (P + I)	5.40 ± 0.51	5.60 ± 0.51	5.80 ± 0.78	6.20 ± 0.78
Percentage of stained areas	22.00 ± 8.73	22.20 ± 7.95	28.70 ± 12.23	29.60 ± 12.65

a Phosphate-buffered saline.

b Platelet rich plasma.

c Conditioned plasma.

d Alpha-smooth muscle actin.

#### 3.3.1 Epithelization score

The epithelization score in the PBS, PRP, bare bead CP, and coated bead CP groups was 2.00 ± 0.47, 2.90 ± 0.31, 3.20 ± 0.42, and 3.60 ± 0.51, respectively. Epithelization rate in all groups was significantly increased compared to the control group (*p* < 0.05), indicating improved epithelial formation in the treatment groups.

#### 3.3.2 Fibroblast score

The fibroblasts score in the PBS, PRP, bare bead CP, and coated bead CP groups was 2.40 ± 0.51, 3.10 ± 0.31, 3.20 ± 0.42, and 3.40 ± 0.51, respectively. Fibroblast analysis showed more significant proliferation and migration of fibroblasts in all groups compared to the control group (*p* < 0.05).

#### 3.3.3 Inflammatory cells infilteration score

Following an injury, an immune response is elicited at the site of the wound, and managing this immune response can aid in the healing process [4]. The inflammatory cells infilteration score in the PBS, PRP, bare bead CP, and coated bead CP groups was 1.90 ± 0.31, 2.50 ± 0.52, 2.90 ± 0.31, and 3.40 ± 0.51, respectively. To investigate the effect of the blood derivatives, inflammatory cell infiltration was evaluated, which showed a higher score and consequently a lower number of inflammatory cells at the wound site in CPs-treated group compared to the control group (*p* < 0.01), as well as a significant difference between the coated bead CP-treated group with the PRP-treated group (*p* < 0.01).

#### 3.3.4 Collagen score

The collagen score in the PBS, PRP, bare bead CP, and coated bead CP groups was 2.20 ± 0.42, 2.30 ± 0.48, 2.80 ± 0.42, and 2.90 ± 0.31, respectively. A significant difference in the synthesis and organization of collagen fibers was observed in the groups of CPs-treated group with the control group (*p* < 0.05) as well as between the coated bead CP-treated group with the PRP-treated group (*p* < 0.05).

#### 3.3.5 New vessels score

Another parameter evaluated was new vessels, which occur in the first days and begin to decrease from 2 to 3 weeks until they reach the level of normal tissue [33,34]. The New vessels score in the PBS, PRP, bare bead CP, and coated bead CP groups was 2.00 ± 0.47, 2.30 ± 0.48, 2.70 ± 0.48, and 3.00 ± 0.47, respectively. The score of this parameter and consequently a fewer new vessel observed in the group treated with CPs-treated group was higher than the control group (*p* < 0.05). There was also a significant difference in its score in the coated bead CP-treated group with the PRP-treated group (*p* < 0.05).

#### 3.3.6 Total score of wound healing

Finally, all the scores collected for different parts of wound healing were summed together, and the overall score for wound healing in all groups was compared (Fig. 4b). The total score of wound healing in the PBS, PRP, bare bead CP, and coated bead CP groups was 10.50 ± 1.17, 13.10 ± 0.99, 14.80 ± 1.22, and 16.30 ± 1.05, respectively. This score indicated a significant difference between the wound healing of all groups with control group (*p* < 0.0001), In addition, a significant difference was between the CPs-treated group with the PRP-treated group (*p* < 0.01) and between the coated bead CP-treated group with bare bead CP-treated group (*p* < 0.01).

### 3.4 Immunohistochemistry

**Fig. 6:**
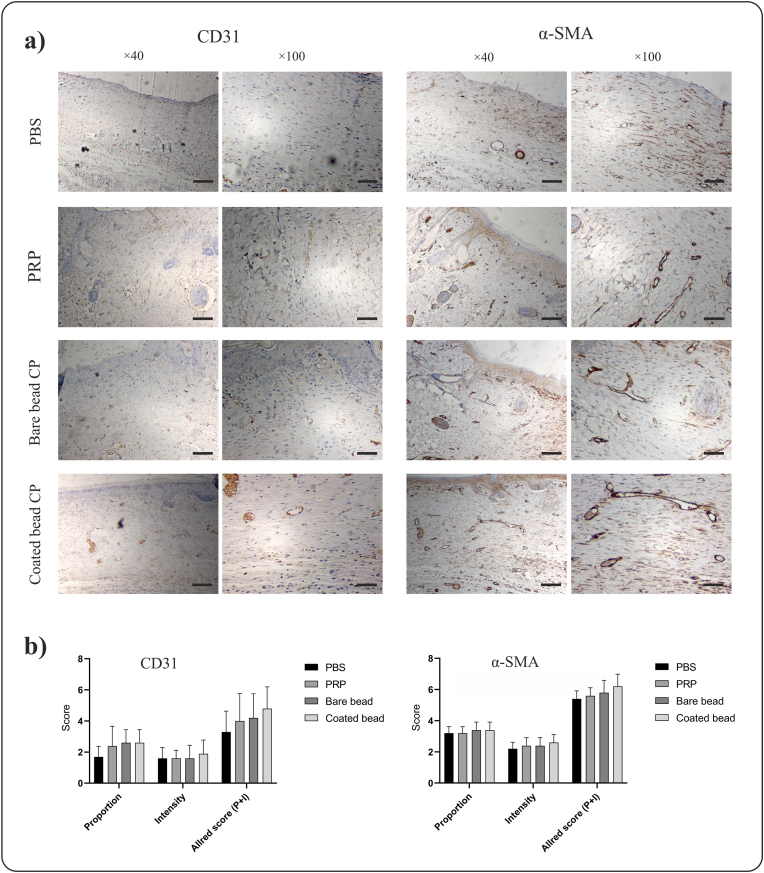
**Immunohistochemical staining of tissue sections at day 14 post wounding from full-thickness wounds in rat**. a) Images of tissue sections of immunohistochemical staining for CD31 and α-SMA in different treatment groups, phosphate buffered saline (PBS), platelet-rich plasma (PRP), bare beads CP, coated beads CP (magnification × 40, scale bars = 200 μm; magnification × 100, scale bars = 100 μm). b) The modified Allred scoring system was used to score these two markers, which showed an increase in the CPs group compared to the other treatment groups, although this increase was not significant (*p* > 0.05).

**Fig. 7:**
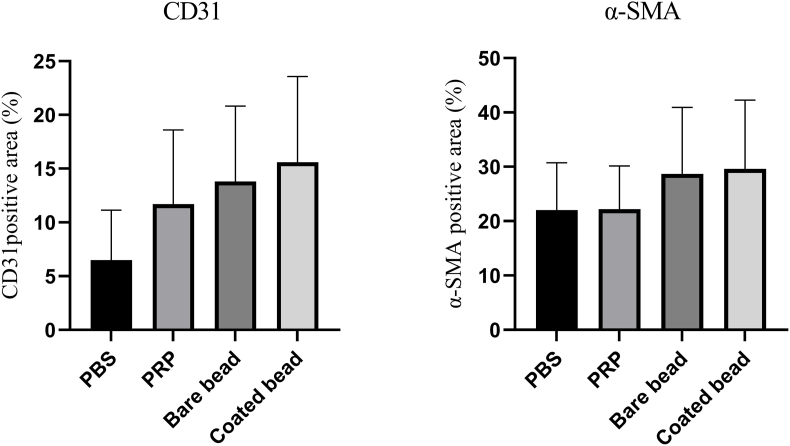
**Percentage of stained areas by immunohistochemical staining**. The percentage quantification of CD31 and α-SMA positive area. The percentage of stained areas was higher in the CPs treated groups, although, this difference was not significant (*p* > 0.05). Data are presented as mean ± SD of full thickens wounds of 5 rats per group and two excisional wounds per rat (n = 10 wounds per group).

Immunohistochemical staining of sections was performed to evaluate CD31 and α-SMA (Fig. 6a). These two markers were scored using the modified Allred scoring system (Fig. 6b) and quantitative analysis using ImageJ software (Fig. 7). Immunohistochemistry staining for CD31 in the PBS, PRP, bare bead CP, and coated bead CP groups yielded the following results: proportion scores of 1.70 ± 0.67, 2.40 ± 1.26, 2.60 ± 0.84, and 2.60 ± 0.84; intensity scores of 1.60 ± 0.69, 1.60 ± 0.51, 1.60 ± 0.84, and 1.90 ± 0.87; and Allred scores of 3.30 ± 1.33, 4.00 ± 1.76, 4.20 ± 1.54, and 4.80 ± 1.39, respectively. In addition, Immunohistochemistry staining for α-SMA in the PBS, PRP, bare bead CP, and coated bead CP groups yielded the following results: proportion scores of 3.20 ± 0.42, 3.20 ± 0.42, 3.40 ± 0.51, and 3.40 ± 0.51; intensity scores of 2.20 ± 0.42, 2.40 ± 0.51, 2.40 ± 0.51, and 2.60 ± 0.51; and Allred scores of 5.40 ± 0.51, 5.60 ± 0.51, 5.80 ± 0.78, 6.20 ± 0.78, respectively. The percentage of stained areas for CD31 in the PBS, PRP, bare bead CP, and coated bead CP groups was 6.49 ± 4.65, 11.70 ± 6.89, 13.80 ± 7.02, and 15.60 ± 7.97, respectively, and for α-SMA, the values were 22.00 ± 8.73, 22.20 ± 7.95, 28.70 ± 12.23, and 29.60 ± 12.65, respectively. The results showed an increase in the score of both markers and percentage of positive stained areas in the groups treated with CPs compared to those treated with PRP and control, but this increase was not significant (*p* > 0.05).

## 4 Discussion

Blood derivatives promote wound healing by containing various growth factors and cytokines [7]. Four key elements in the wound healing process are inflammation, re-epithelialization, wound contraction, and angiogenesis, and enhancing these processes can result in accelerated wound healing [36]. A full-thickness skin wound model was used to investigate the effect of different blood derivatives on wound healing. This study evaluated the progress of wound healing at different time points. The results showed better wound healing in treatment with coated bead CP than in other blood derivatives used. H&E, Masson's trichrome, and immunohistochemistry (CD31 and α-SMA) staining confirmed the study's results.

Platelet-released growth factors such as TGF-β, PDGF, and VEGF are present in various blood derivatives such as PRP, platelet rich fibrin (PRF), and PL. These growth factors play a role in cell proliferation, differentiation, migration, angiogenesis, and tissue repair [7,10,11]. The use of the aforementioned blood derivatives can promote wound healing. In addition, blood derivatives, alone or in combination with other therapies such as stem cells, hydrogels, and acellular dermal matrix (ADM) can help re-epithelialization, cell proliferation, and angiogenesis and promote wound healing [32,[37], [38], [39], [40]]. ACS is another blood derivative that contains various types of cytokines, especially anti-inflammatory cytokines such as IL-1Ra and IL-13 and is used to inhibit inflammation in injuries such as osteoarthritis [19,41]. Inflammation is a phase of wound healing, and regulating it is crucial for tissue repair [1]. IL-13 is a potent cytokine that aids in wound healing and can facilitate the healing process [42]. IL-1Ra is among the anti-inflammatory cytokines, and its suppression is linked to lower inflammation levels and improved wound healing [8,42]. In this study, the growth factors TGF-β, PDGF, VEGF, and the cytokines IL-1β, and IL-1Ra were all found to be at higher concentrations in the CPs. In addition, the concentration of IL-13 was higher in CPs but not statistically significant.

An important factor in proper wound closure is re-epithelialization. Previous studies have shown the effectiveness of blood derivatives alone or in combination with stem cells in wound re-epithelialization [32,36]. Proliferation and differentiation of epidermal cells leads to re-epithelialization, which is carried out with the help of various growth factors such as TGF-β, VEGF, and PDGF [7,43]. In this study, the levels of growth factors, including VEGF, PDGF, and TGF-β, were measured. These growth factors were higher in CPs, especially in coated bead CP, indicating better re-epithelialization and wound healing in rats treated with this blood derivatives.

The first and essential immune mechanism in wound healing is the inflammatory response. If the inflammation at the wound site remains and does not subside, it is detrimental to wound healing. As a result, a balance of inflammatory and anti-inflammatory responses is required for wound healing [1,4]. Studies have shown that blood derivatives such as ACS can reduce the pain and damage of osteoarthritis by reducing IL-1β and improve wound healing by reducing inflammation [8,44]. The CPs produced in this study had high levels of the anti-inflammatory cytokine IL-1Ra and an increased ratio of IL-1Ra/IL-1β, and indicating less infiltration of inflammatory cells and better wound healing.

Collagen deposition is an essential step in wound healing. Collagen deposition and the regular arrangement of collagen fibers in the wound increase the quality of tissue remodeling and reduce the likelihood of scar formation [1,4]. Various studies have shown the effectiveness of PRP alone or in combination treatments in collagen accumulation and shortening wound healing time [32,36,37]. In this study, significant differences in the synthesis and organization of collagen fibers were observed, which indicated an increase in newly formed collagen at the wound site in the treated CPs.

The delivery of nutrients and maintaining oxygen homeostasis is accomplished through blood vessels. The formation of blood vessels in tissue injuries helps in better wound healing, and the quality and quantity of these blood vessels and neovascularization play a decisive role in the quality of wound healing [2]. In this study, the Allred score findings indicated a greater presence of CD31 and α-SMA in the group receiving CPs, even though this enhancement was not statistically significant. The balance between proangiogenic and antiangiogenic factors influences angiogenesis. TGF-β, VEGF, and IL-1β, important in angiogenesis [45], were present in higher concentrations in the produced CPs. The levels of IL-1Ra and the IL-1Ra/IL-1β ratio also increased, potentially inhibiting IL-1β′s effects, which might lead to alterations in angiogenesis. Various studies have utilized human blood derivatives in animal research, demonstrating the efficacy of cross-species treatments [40,43]. These findings suggest that such approaches could hold significant promise for future therapeutic applications. In addition, various studies have been conducted on the effect of recombinant human growth factors on animal model wounds [[46], [47], [48], [49]]. In a study, fibrin combined with different doses of recombinant human vascular endothelial growth factor 165 (rhVEGF165) was used to heal rat wounds [46]. Results showed faster wound healing and more vessels in wounds treated with lower doses of rhVEGF. In the study, demonstrated that neutrophil infiltration reduces while re-epithelialization rises following a skin graft. Moreover, the levels of CD31 and blood vessel formation did not rise during the initial 3 days but increased until day 10, after which the levels began to decline until they matched those of normal skin [34]. Another study demonstrated that laser therapy, when administered at specific doses, can enhance wound healing by promoting fibroblasts proliferation, epithelialization and the formation of new collagen while reducing inflammation. Interestingly, the study observed an initial increase in new blood vessel in the early stages, followed by a decline in vessel by the second week [33]. Furthermore, a study evaluating stem cell therapy, PRP, and their combination in diabetic rat wounds revealed increased expression of CD31 and CD34 markers. However, no significant differences in α-SMA levels were observed across treatment groups [37].Research has demonstrated that utilizing PRP alongside stem cell treatments can enhance angiogenesis in diabetic wounds. In these cases, one challenge in wound care is impaired angiogenesis, and these therapies can promote better angiogenesis and facilitate wound healing [32,37].

## 5 Study limitations

Although the CP product improved wound closure and tissue repair, several limitations may affect generalizability. The short observation period might not capture the full healing response. Chronic wounds, like diabetic ulcers, have distinct pathology that could alter therapeutic responses. While we noted elevated cytokine and growth factor levels, broader profiling may yield deeper mechanistic insights. The CP was tested alone; future studies should evaluate combination therapies, including controlled-release systems or biomaterial scaffolds.

## 6 Conclusion

In summary, this study demonstrated the potential efficacy of the applied CP in promoting wound healing. Its beneficial effects were attributed to enhanced epithelialization, increased fibroblast cells, new collagen formation, and reduced inflammatory cells infiltration. Although this was an animal-based study, further research is warranted to explore the molecular mechanisms underlying these effects and to evaluate the efficacy of this product on various wound types, including diabetic and burn wounds. Additionally, investigations into its use in combination therapies, such as with hydrogels or stem cells, are recommended.

## Data availability statement

This research was conducted at Tarbiat Modares University, and the article's correspondent can provide its data upon reasonable request.

## Ethics approval and consent to participate

Ethics approval for this study was granted by the Tarbiat Modares University Ethics Committee (Ethics No. IR.MODARES.AEC.1403.011)

## Authors' contributions

All authors contributed to the work's conception and main idea. M.Z drafted the main text, figures, and tables and performed laboratory tests and laboratory animal management and studies. S.K and M.Y supervised the work and provided comments and additional scientific information. S.A and M.H·F reviewed and revised the text. B.P performed laboratory tests and data analysis. All authors read and approved the final version of the work to be published.

**Table:** Abbreviation list

Abbreviation	Definition
ACD	Acid citrate dextrose
ACS	Autologous conditioned serum
ADM	Acellular dermal matrix
CP	Conditioned plasma
ELISA	Enzyme-linked immunosorbent assay
FDA	Food and Drug Administration
H&E	Hematoxylin and eosin
IL	Interleukin
IL-1Ra	Interleukin 1 β receptor antagonist
PLA	Polylactic acid
PLA-NP	PLA nanoparticles
PDGF	Platelet-derived growth factor
PPP	Platelet poor plasma
PL	Platelet lysate
PRF	Platelet rich fibrin
PRP	Platelet rich plasma
RBCs	Red blood cells
SMA	Smooth muscle actin
STING	Stimulator of interferon genes
TGF-β	Transforming growth factor beta
VEGF	Vascular endothelial growth factor

## Funding

The current study was supported by the 10.13039/501100008257Tarbiat Modares University, Tehran, Iran [Grant Number: 77913114].

## Declaration of competing interest

Authors declare no conflict of Interests.
